# Predicting Overall Survival in Patients With Multiple Primary Lung Cancer: Nomogram Development and Validation Study

**DOI:** 10.2196/87275

**Published:** 2026-06-26

**Authors:** Wenzhi Luo, Kengliang Rao, Hongjia Chen, Cailian Hu, Shengming Liu, Jing Wang, Dongdong Zhang, Li Chen

**Affiliations:** 1 Department of Pulmonary and Critical Care Medicine, First Affiliated Hospital of Jinan University, 613 Huangpu Avenue West, Tianhe District, Guangzhou, Guangdong, China, 86 02038688888; 2 Department of Pulmonary and Critical Care Medicine, Meizhou People's Hospital, Meizhou, Guangdong, China; 3 Department of Thoracic Surgery, The First Affiliated Hospital of Jinan University, Guangzhou, Guangdong, China

**Keywords:** multiple primary lung cancer, Surveillance, Epidemiology, and End Results database, SEER database, nomogram, survival prediction, overall survival

## Abstract

**Background:**

With the rapid development of medical technology and the emphasis on early lung cancer screening, the detection rate of multiple primary lung cancer (MPLC) has increased in recent years. However, the prognostic determinants and clinical characteristics of patients with MPLC remain poorly characterized.

**Objective:**

This study aimed to develop and validate a nomogram for predicting overall survival (OS) in patients with MPLC using data from the Surveillance, Epidemiology, and End Results database.

**Methods:**

This study was reported in accordance with the TRIPOD (Transparent Reporting of a Multivariable Prediction Model for Individual Prognosis or Diagnosis) guidelines. A cohort of 4177 patients with MPLC (2007‐2015) was obtained from the Surveillance, Epidemiology, and End Results database. The patients were randomly divided into training (n=2923) and validation (n=1254) cohorts at a 7:3 ratio. Backward stepwise Cox regression identified 11 independent risk factors, which were integrated into a nomogram predicting 3-, 5-, and 8-year OS rates.

**Results:**

The nomogram demonstrated superior discriminative ability compared to the American Joint Committee on Cancer staging system, with higher area under the receiver operating characteristic curve values for 3-, 5-, and 8-year OS predictions in both cohorts (training cohort: 0.743, 0.751, and 0.759, respectively; validation cohort: 0.737, 0.734, and 0.695, respectively). Calibration curves and decision curve analysis confirmed its clinical utility.

**Conclusions:**

This study establishes a validated nomogram incorporating clinical and socioeconomic variables to optimize prognostic assessment and personalized treatment planning for patients with MPLC.

## Introduction

Lung cancer remains a leading cause of cancer-related mortality worldwide [1,2]. Multiple primary lung cancer (MPLC), defined as the presence of 2 or more distinct primary lung tumors either synchronously or metachronously, accounts for 3.5% to 15% of newly diagnosed cases [3-6]. With advancements in imaging and an emphasis on early lung cancer screening, the incidence rate of MPLC is increasing worldwide.

MPLC can be classified into metachronous MPLC and synchronous MPLC according to the time of lesion occurrence [7-9]. While advancements in imaging modalities have improved MPLC detection, differentiating it from intrapulmonary metastasis remains clinically challenging, particularly in histologically similar lesions [5-7,10]. Notably, patients with MPLC exhibit significantly better survival outcomes than those with intrapulmonary metastasis, with reported 5-year postresection survival rates ranging from 77.3% to 87% [6,11-17]. Moreover, the prognosis of surgically treated MPLC has been reported to be comparable to that of stage-matched solitary lung cancer [18,19]. Therefore, accurate diagnosis and prognostic assessment of MPLC are of substantial clinical importance.

The TNM staging system plays an important role in treatment selection and prognostic evaluation for patients with lung cancer. However, substantial survival heterogeneity still exists among patients with similar histological subtypes and TNM stages, particularly in MPLC [20]. In MPLC, each lesion is evaluated separately for the tumor, node, and metastasis categories according to current staging recommendations [21]. Furthermore, staging strategies differ among various forms of multifocal lung cancer and among MPLC cases with different radiological manifestations [21]. Thus, patients with MPLC may be inadvertently upstaged to T3 and/or T4 or even M1a, leading to erroneous staging, inaccurate prognosis, and improper treatment [20,22].

Several prognostic nomograms for MPLC have been developed using Surveillance, Epidemiology, and End Results (SEER)–based cohorts [15,23]. However, most existing models primarily focus on clinicopathological variables and provide limited evaluation of socioeconomic factors or the distinct prognostic contributions of the first primary lung cancer (FPLC) and second primary lung cancer (SPLC). In addition, few studies have established long-term survival prediction models for patients with MPLC, and the prognostic applicability of the conventional TNM and American Joint Committee on Cancer (AJCC) staging systems in this population remains limited.

Therefore, this study aimed to identify independent prognostic factors associated with overall survival (OS) in patients with MPLC using the SEER database and develop and validate a prognostic nomogram for predicting 3-, 5-, and 8-year OS. Furthermore, the predictive performance of the nomogram was compared with that of the AJCC staging system to evaluate its potential clinical utility in individualized prognostic assessment and risk stratification for patients with MPLC.

## Methods

### Ethical Considerations

This study analyzed publicly available, deidentified data from the SEER database. The use of this dataset is exempt from institutional review board approval, and the requirement for informed consent was waived for this analysis. The study was conducted in accordance with the Declaration of Helsinki and its subsequent amendments. This study was reported in accordance with the TRIPOD (Transparent Reporting of a Multivariable Prediction Model for Individual Prognosis or Diagnosis) guidelines.

### Patient Selection and Data Processing

From 465,552 lung cancer cases in the SEER database (2007‐2015), a total of 8354 (1.8%) tumor records corresponding to 4177 unique patients were identified as meeting the 2013 American College of Chest Physicians criteria for MPLC. These 4177 patients were included in the final analysis. Exclusion criteria encompassed age below 18 years, diagnosis via autopsy or death certificate, and incomplete variables. Patients with MPLC were identified using *International Classification of Diseases for Oncology, Third Edition*, site codes for lung and bronchus tumors (C33.9 and C34.0-C34.9). The SEER sequence number variable was used to distinguish FPLC from SPLC, with sequence numbers 01 and 02 representing the first and second primary lung tumors, respectively. Patients with sequence numbers of 03 or higher were excluded. Synchronous MPLC and metachronous MPLC were defined according to a diagnostic interval of 6 months or less and more than 6 months, respectively. To minimize potential misclassification of intrapulmonary metastasis, cases with identical histological subtypes were conservatively excluded if tumors were located within the same lobe, accompanied by N1 or N2 disease, or associated with distant metastasis (M1). Eligible patients were randomized into training (70%) and validation (30%) cohorts. The following variables were evaluated: age, sex, AJCC stage, FPLC and SPLC histology, SPLC tumor size, FPLC and SPLC surgery status, average household income, marital status, race, survival status, and survival time. Age (<65 vs ≥65 years), SPLC tumor size (≤3, 3‐5, and >5 cm), and household income (<US $50,000, US $50,000‐80,000, and >US $80,000) were categorized according to the SEER database records for model construction. All TNM stages were uniformly converted to the Eighth Edition of the *AJCC Cancer Staging Manual* using the standard mapping algorithm provided by the SEER database.

### Construction and Validation of the Nomogram

To improve the prediction power of the nomogram, 4177 patients were randomly assigned to the training cohort (n=2923) and the validation cohort (n=1254) at a ratio of 7:3. The prognostic nomogram in this study was developed using the Cox proportional hazards regression model, a conventional statistical method. No machine learning, artificial intelligence, or deep learning algorithms were used for model construction, data analysis, or outcome prediction. Multivariate Cox proportional hazards regression analysis was performed to identify variables that affect OS in the training cohort. Using these predictors of OS, we constructed a nomogram for predicting 3-, 5-, and 8-year survival rates in patients with MPLC.

We subsequently evaluated the discrimination ability of the nomogram by calculating the index of concordance (C-index) and the area under the receiver operating characteristic curve (AUC). The calibration curves show the associations between the predicted and actual probabilities. Both discrimination and calibration were evaluated via bootstrapping with 1000 resamples. The net reclassification improvement (NRI) and integrated discrimination improvement (IDI) were used to compare the accuracy of the nomogram with that of the traditional AJCC staging model. Decision curve analysis (DCA) was used to assess the actual clinical benefits. Finally, AUC values and Kaplan-Meier survival curves were used to compare the nomogram with AJCC staging alone.

### Statistical Analysis

The primary end point was OS, defined as the time from diagnosis of the FPLC to death from any cause or last follow-up. All the statistical analyses were performed via SPSS (version 27.0; IBM Corp) and R (version 4.0.3; R Foundation for Statistical Computing). The statistical tests were 2 sided, and a *P* value of less than .05 was considered to indicate statistical significance.

## Results

### Baseline Characteristics of the Study Participants

**Figure 1.**
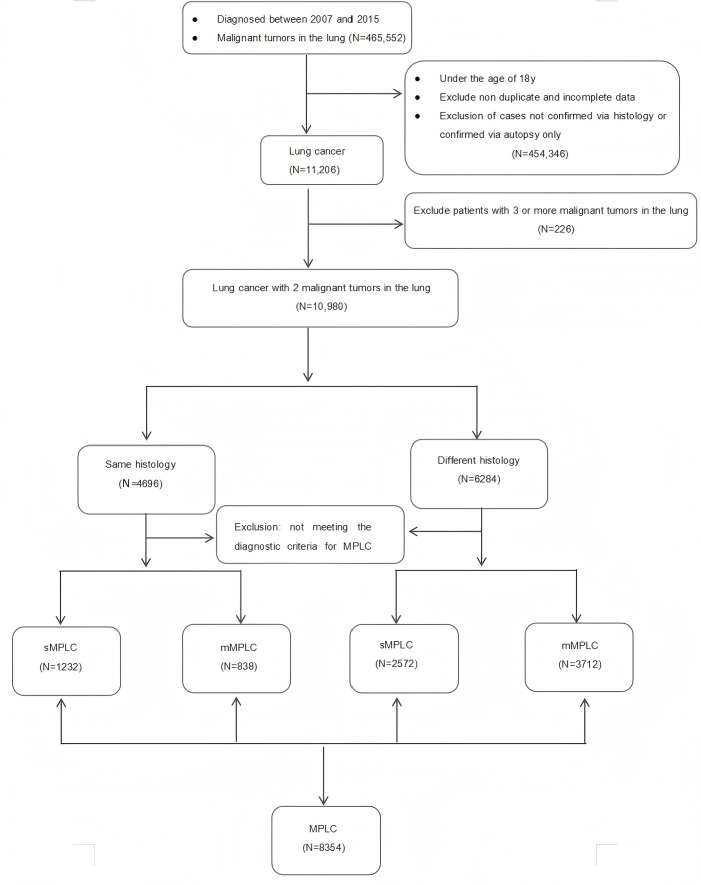
Flow diagram describing the evaluation and selection of patients with multiple primary lung cancer (MPLC). The final cohort consisted of 8354 tumor records representing 4177 unique patients. mMPLC: metachronous MPLC; sMPLC: synchronous MPLC.

We evaluated a total of 4177 patients with MPLC who were randomly divided into a training cohort (n=2923, 70%) and a validation cohort (n=1254, 30%). The selection process of patients with MPLC is shown in Figure 1. The baseline characteristics of the patients in the training and validation cohorts are presented in Multimedia Appendix 1. In the training cohort, most patients (2277/2923, 77.9%) were aged 65 years or older, and 54% (1578/2923) were female. AJCC stage 1 was the most common stage (1459/2923, 49.9%), followed by stage 3 (775/2923, 26.5%). Adenocarcinoma was the predominant histological subtype in both FPLC (1696/2923, 58%) and SPLC (1681/2923, 57.5%). In addition, 61.4% (1796/2923) of patients presented with bilateral lesions. The surgical resection rate was lower for SPLC (1566/2923, 53.6%) than for FPLC (2147/2923, 73.5%). Detailed demographic and clinicopathological characteristics are summarized in Multimedia Appendix 1. During the follow-up period, a total of 2354 deaths were observed, with a maximum follow-up duration of 119 months. For the entire cohort of 4177 patients, the median OS was 68 (95% CI 66‐71) months. The estimated 3-, 5-, and 8-year OS rates were 70.8% (95% CI 69.4%‐72.2%), 55.3% (95% CI 53.7%‐56.9%), and 32.6% (95% CI 30.8%‐34.4%), respectively.

### Univariate and Multivariate Analyses of OS in the Training Cohort

Through univariate and multivariate Cox proportional hazards regression, we identified 11 independent risk factors in the training cohort as follows: age, sex, AJCC stage, histology of FPLC and SPLC, tumor size of SPLC, surgery of both FPLC and SPLC, average household income, marital status, and race, as shown in Table 1.

**Table 1.** Univariable and multivariable analyses of overall survival in the training cohort.

Variable	Univariate analysis		Multivariate analysis	
	HR^a^ (95% CI)	*P* value	HR (95% CI)	*P* value
Age (y)				
<65	Reference	Reference	Reference	Reference
≥65	1.467 (1.295-1.662)	*<*.001	1.416 (1.245-1.610)	*<*.001
Sex				
Male	Reference	Reference	Reference	Reference
Female	0.674 (0.612-0.743)	*<*.001	0.735 (0.662-0.815)	*<*.001
AJCC^b^ stage				
1	Reference	Reference	Reference	Reference
2	1.393 (1.176-1.650)	*<*.001	1.218 (1.025‐1.447)	.03
3	1.642 (1.459‐1.847)	*<*.001	1.353 (1.196‐1.530)	*<*.001
4	2.712 (2.370‐3.104)	*<*.001	2.132 (1.850‐2.456)	*<*.001
Histology of FPLC^c^				
Squamous cell carcinoma	Reference	Reference	Reference	Reference
Adenocarcinoma	0.653 (0.587‐0.726)	*<*.001	0.873 (0.780‐0.976)	.02
SCLC^d^	1.123 (0.928‐1.358)	.23	1.080 (0.888‐1.313)	.44
Others	0.775 (0.613‐0.980)	.03	0.951 (0.751‐1.206)	.68
Histology of SPLC^e^				
Squamous cell carcinoma	Reference	Reference	Reference	Reference
Adenocarcinoma	0.623 (0.557‐0.697)	*<*.001	0.830 (0.737‐0.936)	.002
SCLC	1.745 (1.503‐2.028)	*<*.001	1.618 (1.386‐1.888)	*<*.001
Others	0.968 (0.755‐1.242)	.80	1.150 (0.893‐1.482)	.28
Nodule size of FPLC (cm)				
≤3	Reference	Reference	—^f^	—
3-5	1.246 (1.108‐1.402)	*<*.001	—	—
>5	1.367 (1.168‐1.601)	*<*.001	—	—
Nodule size of SPLC (cm)				
≤3	Reference	Reference	Reference	Reference
3-5	1.989 (1.753‐2.256)	*<*.001	1.630 (1.432‐1.854)	*<*.001
>5	2.641 (2.245‐3.107)	*<*.001	1.983 (1.675‐2.347)	*<*.001
Surgery of FPLC				
No	Reference	Reference	Reference	Reference
Yes	0.568 (0.511‐0.632)	*<*.001	0.869 (0.770‐0.980)	.02
Surgery of SPLC				
No	Reference	Reference	Reference	Reference
Yes	0.453 (0.410‐0.500)	*<*.001	0.633 (0.564‐0.711)	*<*.001
Income (US $)				
<50,000	Reference	Reference	Reference	Reference
50,000-80,000	0.730 (0.657‐0.810)	*<*.001	0.810 (0.727‐0.902)	*<*.001
>80,000	0.699 (0.585‐0.836)	*<*.001	0.835 (0.695‐1.002)	.05
Marital status				
Married	Reference	Reference	Reference	Reference
Divorced or separated	1.108 (0.963‐1.275)	.15	1.147 (0.994‐1.324)	.06
Other	1.157 (1.037‐1.291)	.009	1.142 (1.017‐1.281)	.02
Race				
White	Reference	Reference	Reference	Reference
Black	1.056 (0.889‐1.254)	.54	0.944 (0.792‐1.124)	.52
Others	0.707 (0.554‐0.901)	.005	0.756 (0.591‐0.968)	.03

a HR: hazard ratio.

b AJCC: American Joint Committee on Cancer.

c FPLC: first primary lung cancer.

d SCLC: small-cell lung cancer.

e SPLC: second primary lung cancer.

f Variables excluded from the multivariate analysis.

### Construction and Validation of the Nomogram

**Figure 2.**
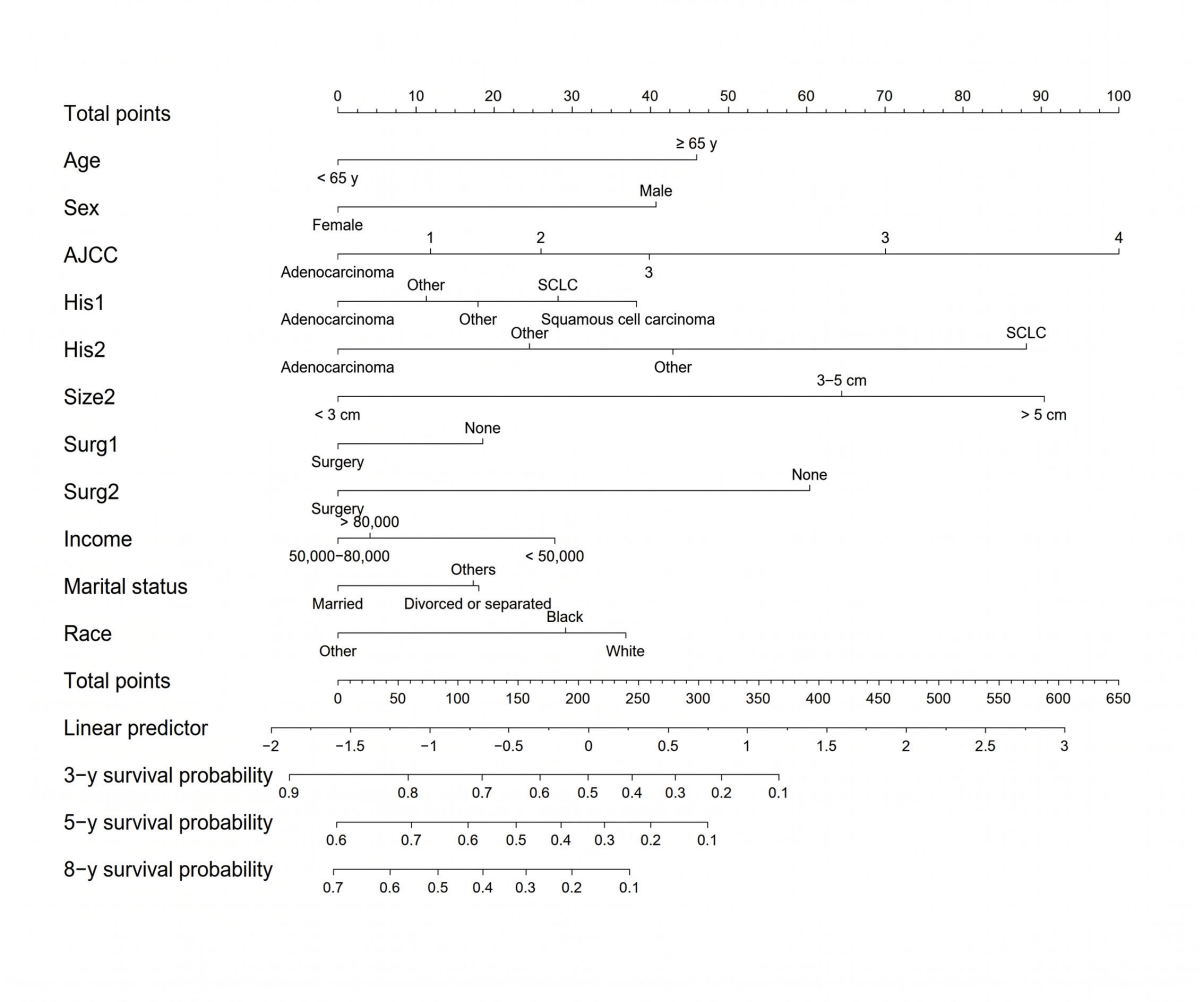
Nomogram predicting 3-, 5-, and 8-year overall survival of patients with multiple primary lung cancer. The factors of age, sex, American Joint Committee on Cancer (AJCC) stage, histology of first primary lung cancer (FPLC; His1) and second primary lung cancer (SPLC; His2), tumor size of SPLC (Size2), surgery for both FPLC (Surg1) and SPLC (Surg2), average household income, marital status, and race were included in the model. SCLC: small-cell lung cancer.

A nomogram was constructed for prediction based on the 11 independent risk factors, which were statistically significant prognostic factors in the Cox proportional hazards model. The nomogram revealed that the AJCC stage had the greatest contribution to survival outcomes, followed by histology, tumor size, and SPLC surgery. We obtained a total score by adding the total points for the score assigned to each variable. The total score corresponded to the prediction of 3-, 5-, and 8-year OS for each patient with MPLC. The higher the score, the worse the survival prognosis. The nomogram is shown in Figure 2.

**Figure 3.**
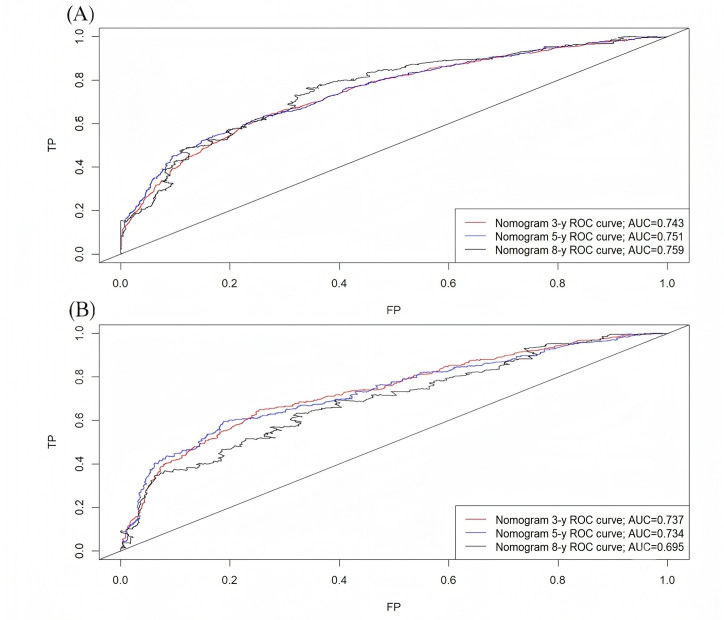
Receiver operating characteristic (ROC) curves of the nomogram for 3-, 5-, and 8-year overall survival prediction: (A) training cohort and (B) validation cohort. AUC: area under the ROC curve; FP: false positive; TP: true positive.

**Figure 4.**
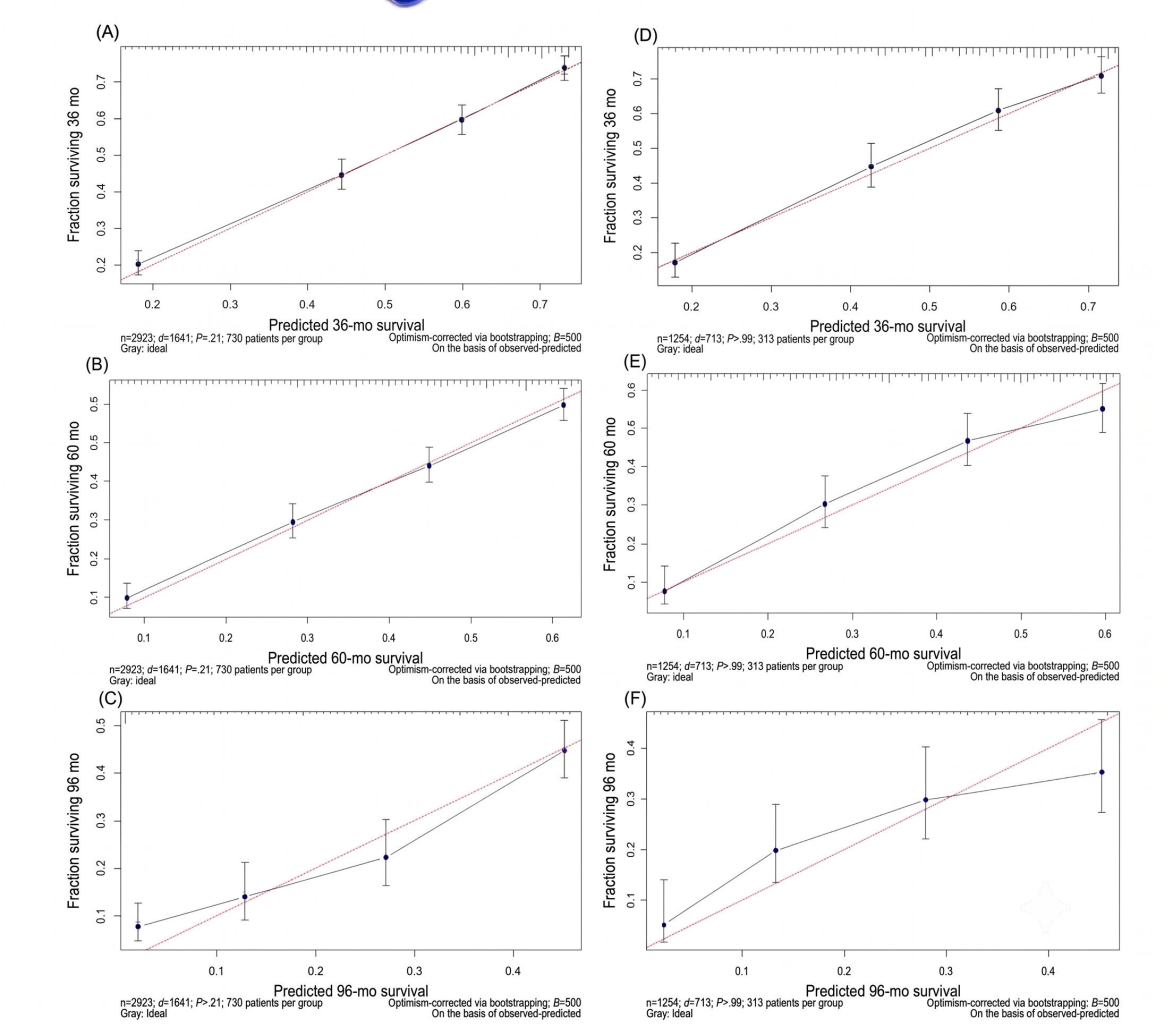
The calibration curves for predicting patient overall survival at 3, 5, and 8 years in the training cohort (A-C) and validation cohort (D-F).

The C-index of the nomogram in the training cohort was 0.702, whereas it was 0.699 in the validation cohort. The AUCs used to predict 3-, 5-, and 8-year OS in the training cohort were 0.743, 0.751, and 0.759, respectively (Figure 3A). In the validation cohort, these values were 0.737, 0.734, and 0.695, respectively (Figure 3B). Furthermore, the calibration curves of the nomogram demonstrated a favorable fit for the 3-, 5-, and 8-year OS probabilities in both the training (Figure 4A-C) and validation (Figure 4D-F) cohorts. There was good consistency between the predicted values and the actual observed values. These findings demonstrated the high accuracy and discriminatory capacity of the nomogram.

**Figure 5.**
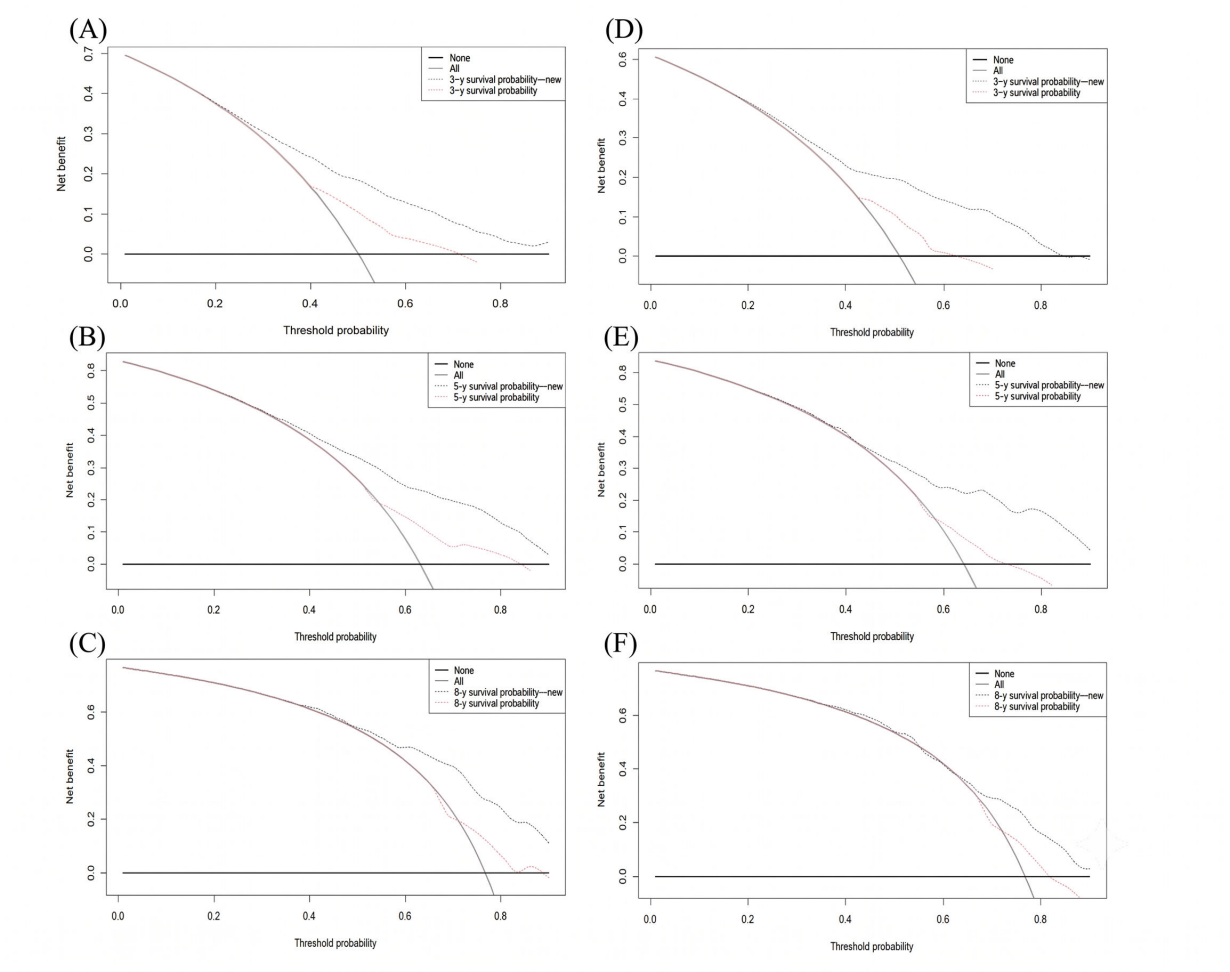
Decision curve analysis for the nomogram and American Joint Committee on Cancer stage of multiple primary lung cancer at 3, 5, and 8 years in the training cohort (A-C) and validation cohort (D-F).

In addition, the NRI values at 3, 5, and 8 years in the training cohort were 0.590 (95% CI 0.505‐0.664), 0.576 (95% CI 0.522‐0.688), and 0.577 (95% CI 0.467‐0.762), respectively. The IDI values for 3-, 5-, and 8-year survival were 0.097, 0.091, and 0.070, respectively, in the training cohort and 0.102, 0.090, and 0.066, respectively, in the validation cohort. All the IDI values were greater than 0 (*P*<.001), indicating a significantly improved prediction performance of the nomogram compared with the AJCC stage model. In both the training cohort and the validation cohort, DCA strongly improved the prediction of 3-, 5-, and 8-year OS (Figure 5).

### Comparison of the Predictive Accuracy Between the Novel Risk Stratification Model and the Conventional Prognostic Model

**Figure 6.**
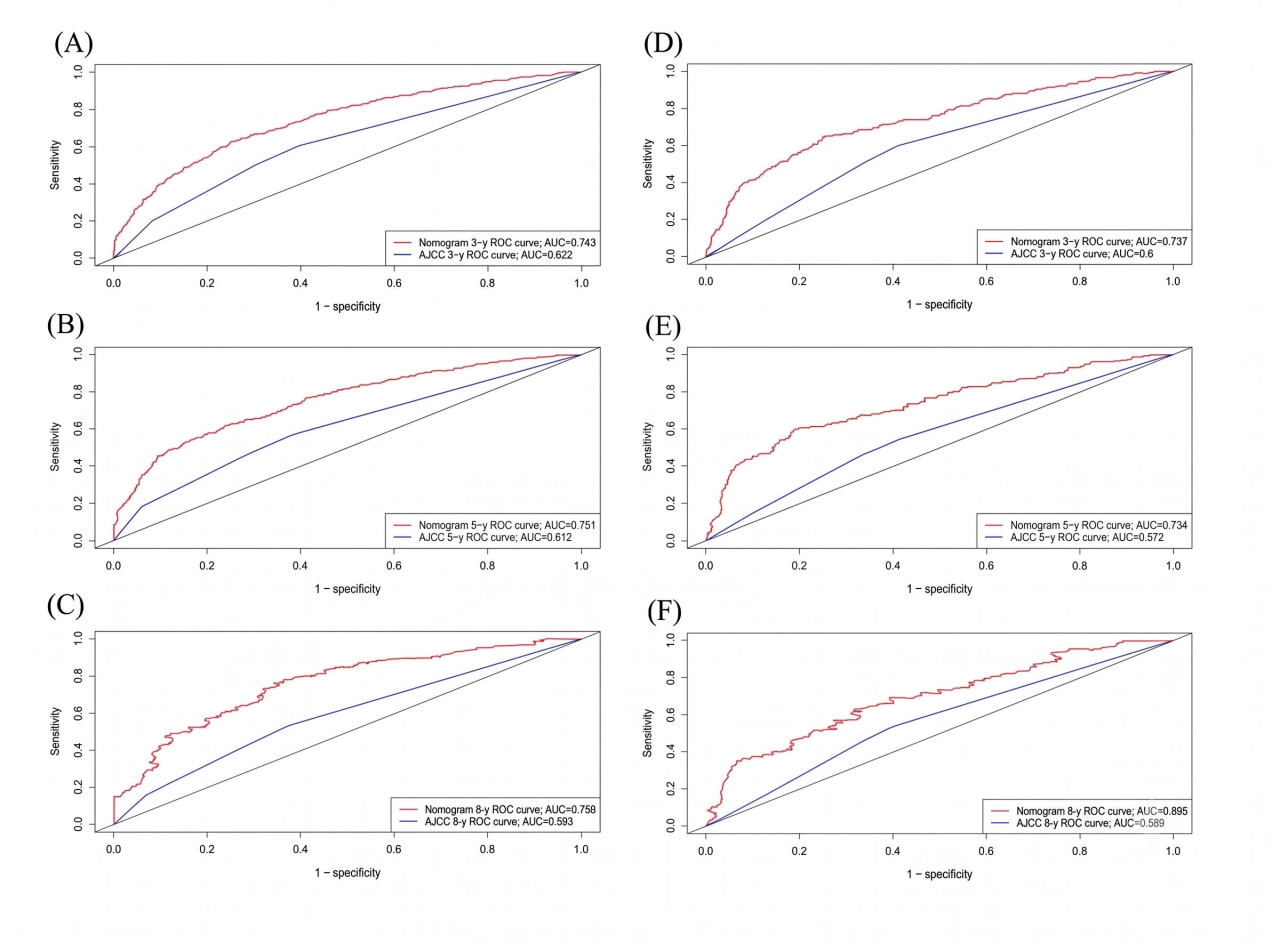
Receiver operating characteristic (ROC) curves of the nomogram and American Joint Committee on Cancer (AJCC) stage prognostic system for 3-, 5-, and 8-year overall survival prediction in the training (A-C) and validation (D-F) cohorts. AUC: area under the ROC curve.

Using AUC values, we compared the accuracy between the nomogram and traditional AJCC staging system. The AUC values of the nomogram and the traditional prediction model were 0.743 vs 0.622, 0.751 vs 0.612, and 0.759 vs 0.583, respectively, in the training cohort (Figure 6A-C) and 0.737 vs 0.60, 0.734 vs 0.572, and 0.695 vs 0.569, respectively, in the validation cohort (Figure 6D-F). We observed that the nomogram had higher AUC values than the AJCC staging model regardless of the prediction of 3-, 5-, and 8-year OS in both the training and validation cohorts.

## Discussion

### Principal Findings

In recent years, the survival prognosis of patients with MPLC has garnered significant research interest. Current evidence indicates that patients with MPLC exhibit substantially improved OS compared to those with intrapulmonary metastasis [5,24,25]. Notably, female patients demonstrate a markedly elevated MPLC incidence (54.2%-73.2%) relative to SPLC (27.9%) or intrapulmonary metastasis (31.6%) [5,20,24], a finding corroborated by our study. However, studies on the prognosis of MPLC are mostly based on small sample sizes, and some important factors affecting the prognosis of patients with tumors are ignored [4-6,12].

In this retrospective study, we developed a Cox proportional hazards regression model and prognostic nomogram for patients with MPLC. Our analysis identified 4 key independent risk factors: AJCC stage, histological subtype of SPLC, secondary tumor size, and surgical management of SPLC. Additionally, female sex, higher socioeconomic status, and race emerged as favorable prognostic indicators. On the basis of these factors, a nomogram predicting the probability of survival calibrated well and was a good measure of discrimination.

Compared with previously published SEER-based prognostic nomograms for MPLC, our model incorporated both clinicopathological and socioeconomic variables and separately evaluated the histological characteristics of FPLC and SPLC. In addition, this nomogram provided simultaneous prediction of 3-, 5-, and 8-year OS and demonstrated improved predictive performance compared with the conventional AJCC staging system. These findings suggest that the model may offer additional value for individualized prognostic assessment in patients with MPLC.

While the AJCC staging system partially predicts lung cancer outcomes, its prognostic utility in MPLC requires refinement. A prior study by Tanvetyanon et al [15] highlighted sex, younger age, smaller tumor size, absence of nodal involvement, and bilateral presentation as significant prognosticators in MPLC. Our findings align with these observations and further establish AJCC stage as the most influential prognostic determinant [26]. Notably, Yu et al [18] identified tumor size as the sole prognostic factor in node-negative patients with MPLC, whereas subsequent studies [15,27-29] confirmed that survival outcomes in early-stage MPLC (T1-T2) parallel those of solitary lung nodules, significantly surpassing stage 3 and 4 non–small-cell lung cancer outcomes based on the Eighth Edition of the *AJCC Cancer Staging Manual*.

Histological characteristics, particularly those of SPLC, significantly impacted survival outcomes. Adenocarcinoma histology was independently correlated with improved prognosis, consistent with prior reports [15,30]. However, neither the aforementioned study by Tanvetyanon et al [15] nor a study by Song et al [23] determined that the histology from FPLC or SPLC had a greater impact on the prognosis of MPLC. We conducted an independent analysis of the histology of 2 primary lung cancers in MPLC. The nomogram revealed that the contribution of histological variables to SPLC was significantly greater than that to FPLC. The survival prognosis of patients with MPLC was related mainly to the histological type of SPLC.

In clinical evaluation, distinguishing between synchronous and metachronous MPLC is of great significance. Although the SEER database does not provide variables that can be used to clearly define the sequence of onset, the proportion of bilateral lesions in the training cohort of this study was as high as 61.4% (1796/2923), suggesting that this patient population was predominantly characterized by synchronous or near-synchronous onset. Regarding pathological consistency, the model in this study included the histological types of FPLC and SPLC as 2 independent variables in the analysis rather than simply combining them into a binary “pathologically consistent/inconsistent” indicator. This design allows the line plot to more fully reflect the prognostic value of the corresponding histological types of the 2 tumors, particularly highlighting the importance of the histological subtype of SPLC for prognosis without being confounded by whether the histologies of the 2 primary cancers were consistent. In the future, it will be necessary to further validate the results of this study in different subgroups based on more detailed information regarding the sequence of onset.

Surgical intervention for SPLC conferred superior prognostic benefits compared to FPLC management. Cox regression analysis revealed substantially worse outcomes in nonsurgical cohorts, potentially attributable to misdiagnosis as metastatic or recurrent disease leading to therapeutic delays. Current consensus emphasizes surgical resection as the main approach to MPLC management [3], supported by evidence of enhanced long-term survival vs stage 4 non–small-cell lung cancer or metastatic disease [30,31]. The research by Stella et al [31] further advocates for aggressive surgical approaches for eligible patients with adequate cardiopulmonary reserve in MPLC or multiple lesions. Moreover, the differences in imaging assessment, inconsistencies in pathological diagnostic criteria, interobserver variability, variability in tissue sampling methods, lack of standardized definitions for distinguishing MPLC from intrapulmonary metastasis, and differences in diagnostic workup across institutions or time periods could bias the diagnosis of MPLC considering that current guidelines focus mainly on the recurrence of intrapulmonary metastasis rather than the development of MPLC. Establishing a risk profile for the survival prognosis of patients with MPLC would be beneficial.

In our study, being older; male; from a low-income background; divorced, separated, or single; or White were poor prognostic factors for MPLC. MPLC is common in patients aged 65 years and older, and the prognosis of patients with MPLC becomes worse with age, similar to most malignant tumors. Although there were more female patients than male patients in this study, the risk of death in female patients is lower than in male patients, which is due to the emotional and psychological stress and hormone levels of female patients. In terms of economic value, low-income individuals do not pay attention to the disease and cannot afford medical expenses, which is an important reason for the high risk of death of patients with MPLC among low-income individuals. Married patients may have better survival outcomes than other patients. However, with respect to the impact of race on the prognosis of MPLC, because the information was collected from the SEER database, White patients comprised most of the data, and more real-world data are needed to support the conclusion that White individuals have a high incidence of MPLC.

According to the results of this study, the nomogram demonstrated good distinguishing ability and high accuracy, with training and validation cohort C-indexes of 0.702 and 0.699, respectively. Time-dependent AUC values exceeded 0.70 for 3-, 5-, and 8-year survival predictions. The calibration curve was close to the reference line and fell along the diagonal line of 45 degrees, which shows that the nomogram was in good agreement with the actual results and that the degree of fit was high. The NRI and IDI values were greater than 0, indicating that the nomogram had improved prediction performance over traditional AJCC staging. The DCA curve demonstrated that the nomogram yielded a high net benefit compared to alternative strategies in both the training and validation cohorts, indicating that it meets the practical needs of clinical decision-making and can be widely used in clinical analysis. This nomogram provides continuous estimates of survival probability rather than assigning patients into predefined risk categories. Preserving continuous probability estimation avoids the loss of prognostic information associated with arbitrary cutoff selection and enables more individualized risk assessment. The continuous scores generated by the model may assist clinicians in personalized follow-up surveillance, prognostic evaluation, and clinical risk stratification for patients with MPLC. For example, patients with progressively increasing model-derived risk scores may benefit from more intensive follow-up monitoring, thereby avoiding the limitations of the traditional “one-size-fits-all” approach inherent in coarse risk stratification systems. Therefore, we frame this nomogram as a clinical decision support tool for individualized prognostic assessment and risk stratification rather than as a stand-alone prognostic classifier.

### Limitations

This study has certain limitations, and caution should be exercised when interpreting and applying its findings. First, as this was a retrospective analysis based on the SEER database, some clinical information was incomplete, which may introduce selection bias; key prognostic factors such as smoking, alcohol consumption, family history of cancer, genotyping, detailed imaging characteristics, and laboratory test results were missing, making it impossible to adjust for these as confounding factors in the analysis. Second, due to limitations in the database itself, treatment details were limited. Data on specific regimens for radiotherapy, chemotherapy, targeted therapy, and immunotherapy were lacking, making it difficult to accurately assess the impact of different treatments on patient prognosis. Third, this study included only cases of dual primary lung cancer and did not include cases with 3 or more primary tumors; therefore, the conclusions cannot be extrapolated to a broader population of patients with MPLC. Fourth, the constructed nomogram has not yet undergone external validation in independent cohorts from other medical institutions; its generalizability across different populations and clinical settings requires further verification. Fifth, this study used OS as the primary end point rather than tumor-specific survival; non–cancer-related deaths may introduce confounding factors into the survival analysis results. Additionally, the model’s C-index was approximately 0.70, indicating moderate but clinically meaningful discriminatory ability rather than perfect prediction accuracy. Therefore, this nomogram should be considered a clinical decision support tool for individualized prognostic assessment and risk stratification rather than an independent prognostic classifier. In summary, the model may assist clinicians in developing individualized follow-up and treatment strategies, but it should not be used alone for final clinical decision-making.

### Conclusions

In summary, we constructed a nomogram for patients with MPLC using the SEER database that has good ability to predict the individual survival of patients with MPLC more efficiently, accurately, and easily. This information can help clinicians evaluate the prognosis of patients better than before and provide individualized treatment.

## Supplementary material

10.2196/87275 Multimedia Appendix 1 Baseline characteristics of the patients in the training and validation cohorts.

## Abbreviations

AJCC: American Joint Committee on Cancer
AUC: area under the receiver operating characteristic curve
DCA: decision curve analysis
FPLC: first primary lung cancer
IDI: integrated discrimination improvement
MPLC: multiple primary lung cancer
NRI: net reclassification improvement
OS: overall survival
SEER: Surveillance, Epidemiology, and End Results
SPLC: second primary lung cancer
TRIPOD: Transparent Reporting of a Multivariable Prediction Model for Individual Prognosis or Diagnosis
