# Supplementary material for: Predicting Overall Survival in Patients With Multiple Primary Lung Cancer: Nomogram Development and Validation Study
Source: JMIR Cancer. 2026 Jun 26;12:e87275. doi: 10.2196/87275 (PMC13309065; doi:10.2196/87275)
Supplement: Multimedia Appendix 1 [file cancer-v12-e87275-s001.pdf]

Table 1. Demographic and clinical characteristics of patients in the training and validation cohorts

Variables, n(%)	Training cohort (n=2923)	Validation cohort (n=1254)
Age, years		
＜65	646（22.1）	283（22.6）
≥65	2277（77.9）	971（77.4）
Sex		
Male	1345（46.0）	573（45.7）
Female	1578（54.0）	681（54.3）
AJCC stage		
I	1459（49.9）	618（49.3）
II	280（9.6）	113（9.0）
III	775（26.5）	357（28.5）
IV	409（14.0）	166（13.2）
Laterality		
Unilateral	1127（38.6）	554（44.2）
Bilateral	1796（61.4）	700（55.8）
His1		
Squa	893（30.6）	366（29.2）
Aden	1696（58.0）	685（54.6）
Sclc	196（6.7）	109（8.7）
0thers	138（4.7）	94（7.5）
His2		
Squa	789（27.0）	326（26.0）
Aden	1681（57.5）	735（58.6）
Sclc	341（11.7）	141（11.3）
0thers	112（3.8）	52（4.1）
Size1, cm		
≤3	1997（68.3）	839（66.9）
3-5	627（21.5）	280（22.3）
>5	299（10.2）	135（10.8）
Size2, cm		
≤3	2272（77.7）	992（79.1）
3-5	437（15.0）	177（14.1）
>5	214（7.3）	85（6.8）
Surg1		
None	776（26.5）	319（25.4）
Yes	2147（73.5）	935（74.6）
Surg2		
None	1357（46.4）	573（45.7）
Yes	1566（53.6）	681（54.3）
Rad1		
None/Unknown	2198（75.2）	942（75.1）
Yes	725（24.8）	312（24.9）
Rad2		

None/Unknown	1916（65.5）	821（65.5）
Yes	1007（34.5）	433（34.5）
Chem1		
None/Unknown	2052（70.2）	863（68.8）
Yes	871（29.8）	391（31.2）
Chem2		
None/Unknown	2065（70.6）	871（69.5）
Yes	858（29.4）	383（30.5）
Income, $		
＜50000	918（31.4）	393（31.3）
50000-80000	1713（58.6）	732（58.4）
＞80000	292（10.0）	129（10.3）
Marital status		
Married	1630（55.8）	677（54.0）
Div/Sep	431（14.7）	171（13.6）
Others	862（29.5）	406（32.4）
Race		
White	2528（86.5）	1092（87.1）
Black	240（8.2）	117（9.3）
Others	155（5.3）	45（3.6）

“His 1” = Histology of FPLC; “His 2” = Histology of SPLC; “Aden” = adenocarcinoma; “Squa” = squamous cell carcinoma; “Sclc” = small cell lung cancer;“Size 2” = Nodule size of SPLC; “Surg 1” = surgery of FPLC; “Surg 2” = surgery of SPLC; “Rad 1” = radiation therapy of FPLC; “Rad 2” = radiation therapy of SPLC; “Chem 1” = chemotherapy of FPLC; “Chem 2” = chemotherapy of SPLC; “Div/Sep” = divorce or separation.
